# Modulation of Conductivity and Contact Resistance of RuO_2_ Nanosheets via Metal Nano-Particles Surface Decoration

**DOI:** 10.3390/nano11092444

**Published:** 2021-09-19

**Authors:** Jongwon Kim, Seonhye Youn, Ju Young Baek, Dong Hwan Kim, Sumin Kim, Wooyoung Lee, Hee Jung Park, Juyoung Kim, Dong Won Chun, Sang-Shik Park, Jong Wook Roh, Jeongmin Kim

**Affiliations:** 1 Division of Nanotechnology, DGIST, 333 Techno Jungang-daero, Daegu 42988, Korea; jwkp9605@knu.ac.kr (J.K.); jooyeong@dgist.ac.kr (J.Y.B.); Kimdhwan@dgist.ac.kr (D.H.K.); 2 School of Nano Materials Engineering, Kyungpook National University, Sangju 37224, Korea; parkss@knu.ac.kr; 3 Department of Materials Science and Engineering, Yonsei University, 50 Yonsei-ro, Seoul 03722, Korea; seonhye0718@yonsei.ac.kr (S.Y.); suminkim@yonsei.ac.kr (S.K.); wooyoung@yonsei.ac.kr (W.L.); 4 Department of Materials Science and Engineering, Dankook University, 119 Dandae-ro, Cheonan 31116, Korea; parkjang@dankook.ac.kr; 5 Center for Energy Materials Research, Korea Institute of Science and Technology, Seoul 02792, Korea; jykim1299@kist.re.kr (J.K.); chundream98@kist.re.kr (D.W.C.)

**Keywords:** two-dimensional materials, RuO_2_, nanosheets, surface charge transfer, electrical conductivity, contact resistance

## Abstract

We studied the variation in electrical conductivity of exfoliated RuO_2_ nanosheets and the modulation in the contact resistance of individual nanosheet devices using charge transfer doping effects based on surface metal nanoparticle decorations. The electrical conductivity in the monolayer and bilayer RuO_2_ nanosheets gradually increased due to the surface decoration of Cu, and subsequently Ag, nanoparticles. We obtained contact resistances between the nanosheet and electrodes using the four-point and two-point probe techniques. Moreover, the contact resistances decreased during the surface decoration processes. We established that the surface decoration of metal nanoparticles is a suitable method for external contact engineering and the modulation of the internal properties of nanomaterials.

## 1. Introduction

Numerous researchers have focused on studying two-dimensional (2D) materials since Novoselov and Geim first demonstrated the unique physical properties of graphene, which is a single layer of carbon [1,2]. Two-dimensional materials find application in a wide range of fields, such as atomic electronics [3,4], photonics [5,6], and flexible electronics [7,8]. Therefore, researchers have studied 2D materials extensively, using various layered materials, such as transition metal dichalcogenides [9,10,11,12,13], black phosphorous [14,15,16], and hexagonal boron nitride [17,18], and 2D metal oxides have potential applications in fields such as catalysis [19], solar cells [20], supercapacitors [21], and energy storage devices [22]. Ruthenium oxide (RuO_2_) nanosheets have a rutile structure and metallic characteristics as a bulk crystal. Therefore, RuO_2_ nanosheets can be used as a flexible transparent conducting material with high thermodynamic stability [23,24]. Researchers observed sheet resistance in RuO_2_ films consisting of exfoliated nanosheets in studies on capacitors [25], conducting additives [26], and chemical sensors [27]. The crystal structure and physical properties of individual monolayer RuO_2_ nanosheets were first studied using the potassium-intercalated RuO_2_ [28]. Recently, researchers have demonstrated methods to increase electrical conductivity [29] and modulate the thermoelectric properties [30] of the exfoliated RuO_2_ nanosheets through surface decoration treatments using Ag nanoparticles. This established the potential application of RuO_2_ nanosheets as a flexible transparent conducting material.

We studied the electrical transport properties of individual RuO_2_ nanosheet devices that underwent surface treatment with metal nanoparticles to enhance their electrical conductivity. We measured the variations in electrical conductivity as a result of the surface charge transfer doping of Cu and Ag nanoparticles in monolayer and bilayer RuO_2_ nanosheets as a function of temperature. Moreover, we performed a quantitative analysis of the contact resistances between the nanosheet and electrodes, which had been overlooked in past studies, using two different measurement configurations, with four-terminal devices. We demonstrated a methodology to modulate the contact properties of devices and materials using RuO_2_ nanosheets as a conductive material.

## 2. Materials and Methods

### 2.1. Materilas

**Figure 1:**
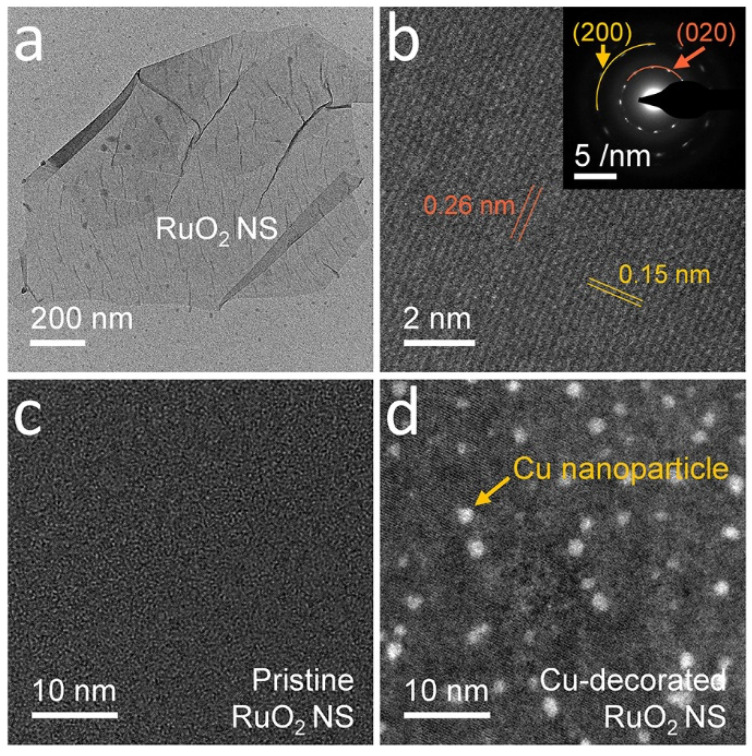
Exfoliated RuO_2_ nanosheet and metal nanoparticle decoration. (**a**) Transmission electron microscopy (TEM) and (**b**) scanning transmission electron microscopy (STEM) images of an exfoliated RuO_2_ nanosheet. The inset image of (**b**) illustrates an electron diffraction (ED) pattern of the nanosheet. (**c**,**d**) Low-magnitude TEM images of a RuO_2_ nanosheet (**c**) before, and (**d**) after, the surface decoration with Cu nanoparticles.

We mixed potassium carbonate (K_2_CO_3_ from Wako Pure Chemical Corporation, Osaka, Japan) and ruthenium dioxide (RuO_2_ from Wako Pure Chemical Corporation) powders in a 5:8 ratio and pelletized the mixture. The pelletized mixture was heated to 850 °C for one day in a N_2_ atmosphere, followed by stirring in water at room temperature for one day. The potassium ruthenates (K_x_RuO_2_) were filtered and stirred into 1 M hydrochloric acid (HCl from Wako Pure Chemical Corporation) aqueous solution for 3 days. H^+^ ions substituted K^+^ during this process, resulting in hydrogen ruthenates (H_x_RuO_2_). We mixed H_x_RuO_2_ (4 g) with tetrabutylammonium hydroxide (TBAOH, Sigma-Aldrich, St Louis, USA) in water (1 L) and stirred the aqueous solution at room temperature for 14 days. The RuO_2_ nanosheet was exfoliated and stabilized using tetrabutylammonium ions (TBA^+^). Figure 1a illustrates a low-magnitude transmission electron microscopy (TEM, FEI Titan 80-300) image of a 2D nanosheet fabricated using the proposed method. We performed scanning transmission electron microscopy (STEM, FEI Titan 80-300) to study the atomic arrangement of the RuO_2_ nanosheet (Figure 1b). The interplanar d-spacing in the electron diffraction (ED) pattern and inter-atomic distance in STEM were equivalent to the d-spacing between the monolayer RuO_2_ nanosheets obtained from our calculations [28]. The synthesis and exfoliation of RuO_2_ nanosheets were described in detail elsewhere [28,29,31].

### 2.2. Metal Nanoparticle Surface Decorations

We employed surface charge transfer, using the surface decoration of metal nanoparticles, to modulate the electrical transport properties of RuO_2_ nanosheets [29,30]. Copper (Cu) nanoparticles were decorated on the nanosheets using 0.05 M Cu acetate (Cu(OOCCH_3_)_2_ > 99.999%, Alfa Aesar, Haverhill, MA, USA) at room temperature for one day and washed with deionized water to remove the residual particles. The nanoparticle decorated RuO_2_ nanosheet was immersed in 0.05 M sodium borohydride (NaBH_2_ > 99.99, Aldrich, St Louis, MO, USA) for 2 min to reduce the copper oxide (CuO_x_) formed as a by-product of the surface decoration process using Cu acetate. Figure 1c,d illustrate the surface of a RuO_2_ nanosheet before and after the Cu nanoparticle decoration process. We carried out the subsequent Ag nanoparticle decoration following the same processes, using 0.05 M Ag acetate (CH_3_COOAg > 99%, Aldrich, St Louis, MO, USA) for additional carrier density modulations of the RuO_2_ nanosheets.

### 2.3. Device Fabrication

**Figure 2:**
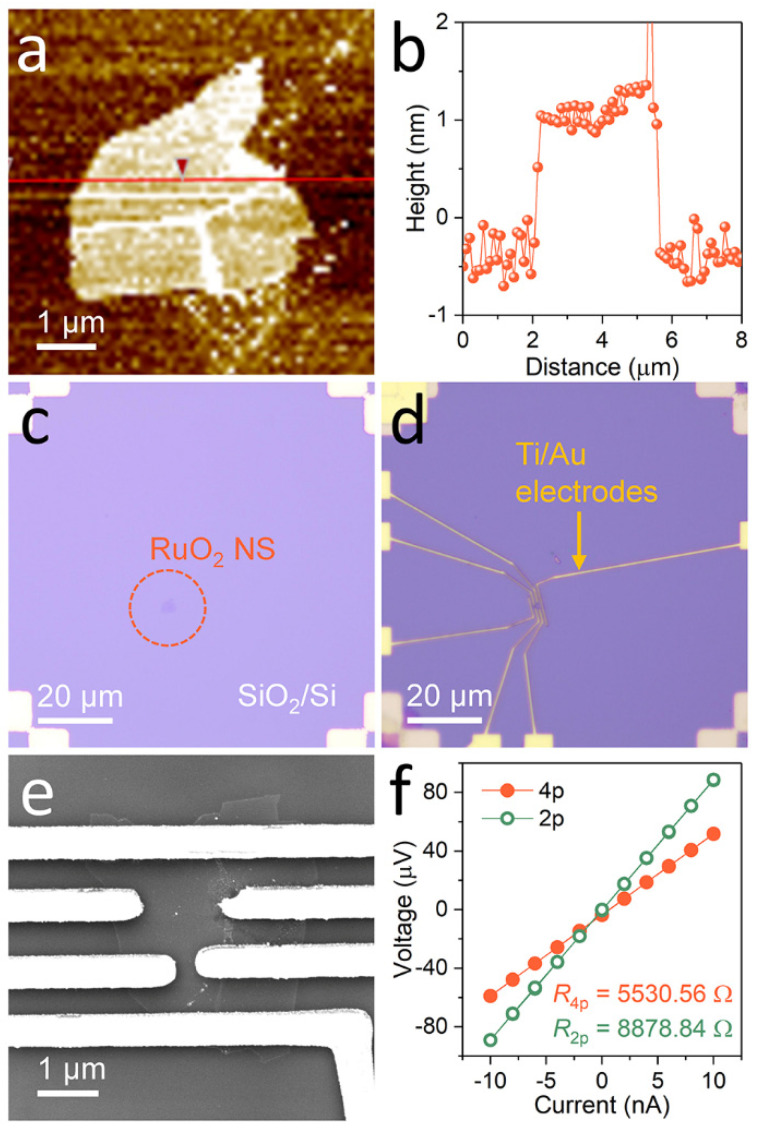
Device fabrication and four-point probe measurement. (**a**,**b**) Atomic force microscopy (AFM) image and height profile of a monolayer RuO_2_ nanosheet. (**c**,**d**) Optical microscopy (OM) images of the nanosheet (**c**) before and (**d**) after metallization process. (**e**) Scanning electron microscopy (SEM) image of the nanosheet device with four terminals. (**f**) *I*–*V* curves obtained from the monolayer RuO_2_ nanosheet using the four- (closed orange circles) and two-point probe (open green circles) measurement techniques. All images and data were obtained from the same nanosheet.

We diluted the chemically exfoliated RuO_2_ solution in deionized (DI) water in a 1:20 ratio. Before dispersing the diluted RuO_2_ nanosheets, we used the O_2_ plasma method (COVANCE, Femto Science Inc.) for hydrophilic surface treatment on thermally oxidized silicon (SiO_2_/Si) substrates. Moreover, the alignment marks were patterned using the photolithography process (MDA-400S, Midas System) during our experiment. We determined the thickness of the nanosheets using atomic force microscopy (AFM, XE-150, Park Systems) on the SiO_2_/Si substrates to test the monolayer and bilayer RuO_2_ nanosheets (Figure 2a,b) [30]. The nanosheets dispersed on the substrate were patterned for transport measurements using electron-beam lithography (VEGA 3, Tescan and NPGS, JC Nabity Lithography Systems) and the lift-off process (Figure 2c,d). The open patterns were exposed to inductively coupled Ag plasma for 3 min, followed by deposition of Ti (10 nm)/Au (150 nm) using an ultra-high vacuum etching and sputtering system (custom-made) to improve the electrical contact between the nanosheet and electrodes [32].

### 2.4. Measurement Techniques

Figure 2e illustrates a scanning electron microscopy (SEM, JEOl-7800F, JEOL Ltd.) image of a four-terminal device with individual monolayer RuO_2_ nanosheets. We measured the resistances of the individual monolayer RuO_2_ nanosheets using four-point and two-point probe measurement techniques at constant current (2182 Nanovoltmeter and 236 Source Measure Unit, Keithley) to obtain their electrical transport properties. In the four-point probe measurement, the voltage drop due to contact resistance was excluded by physically separating the voltage measurement circuit (inner two electrodes) from the applied current circuit (outer two electrodes). On the other hand, the two-probe resistance value includes the contact and measurement circuit resistance because the voltage drop was measured across the overall circuit [32,33]. Figure 2f illustrates the *I*–*V* curves and resistances of a monolayer RuO_2_ nanosheet obtained using two different measurement configurations. We calculated the electrical conductivity of the nanosheets using the following formula: *σ* = *L/*(*R·w·t*), where *σ*, *L*, *R*, *w*, and *t* are the electrical conductivity, length, resistance, width, and thickness, respectively. Moreover, we used a closed-cycle cryostat (X-1AL, ARS) under high-vacuum conditions to measure all transport properties, including the temperature dependence [33].

## 3. Results and Discussion

### 3.1. Increase in Electrical Conductivity

**Figure 3:**
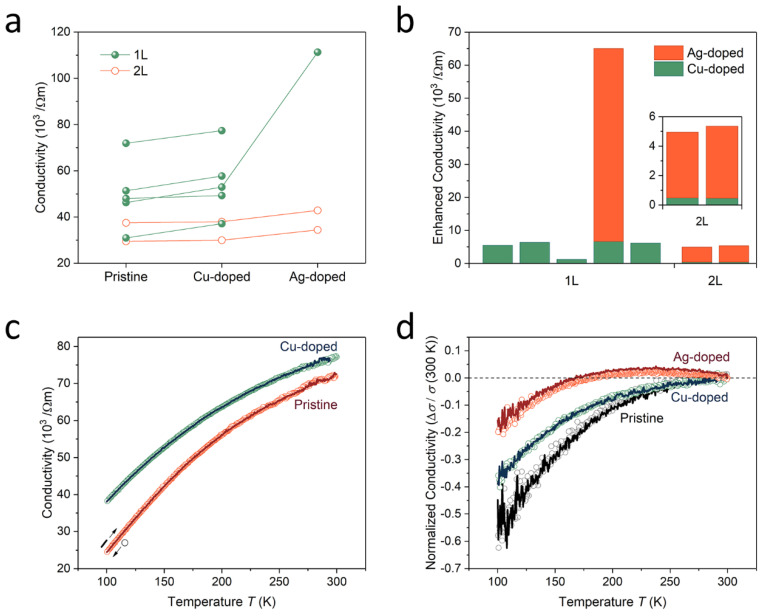
Enhancement of electrical conductivity. (**a**) Increasing the electrical conductivity of RuO_2_ nanosheets using surface decoration with Cu and Ag nanoparticles. The closed green and open orange circles represent the conductivity values obtained from monolayer and bilayer nanosheets, respectively. (**b**) Magnitude of the conductivity gain in the Cu (green) and Ag (orange) treatments. The small conductivity gains of Cu-doped bilayers can be confirmed in the inset. (**c**) Temperature dependent electrical conductivities before (orange) and after (green) the Cu treatment obtained from a monolayer nanosheet using the four-probe measurement technique. The open circles and solid lines indicate the data measured during temperature ramp-down and ramp-up, respectively. (**d**) Direct comparison of the temperature dependencies in pristine (black), Cu-doped (green), and Ag-doped (orange) states using the normalized conductivities, based on room temperature values.

Figure 3a demonstrates the variations in the electrical conductivity of the pristine RuO_2_ nanosheets due to the surface decoration treatment using Cu-nanoparticles followed by Ag-nanoparticles. We tested five-different monolayer and two bilayer nanosheets using the four-point probe measurement technique to determine the relationship between the surface doping process and the nanosheet thickness [28]. Although we observed differences in the conductivity of the nanosheets, the distributions of the conductivity values were consistent with the distributions observed in past studies using single-crystalline RuO_2_ nanosheets [29]. Furthermore, the increase in the magnitude of conductivity was approximately constant with the nanosheets with the same number of layers, except for one nanosheet. The deviations in enhanced conductivity were equal to 0.08 and 0.03 (10^3^ Ωm^−1^) for the 4-monolayer and 2-bilayer nanosheets, respectively (Figure 3b). The impact of Ag doping on the electrical conductivity of the nanosheets was approximately 10-times stronger than the impact of Cu doping in the both monolayer and bilayer nanosheets, but was weaker than the impact of Ag decoration without Cu decoration observed in past studies [29]. This was because the majority of the surface area of the nanosheets was covered with pre-decorated Cu nanoparticles, leaving insufficient surface area for decoration with Ag nanoparticles, which have a higher doping efficiency than Cu nanoparticles. In both metal nanoparticle doping processes, the conductivities of the monolayers were 10-times higher compared to the conductivities of the bilayers. This result agreed with the results from past studies [29].

Figure 3c demonstrates the change in electrical conductivity before and after the Cu decoration process as a function of the temperature obtained from the monolayer nanosheet, with the highest room-temperature conductivity when using the four probes. The increase in conductivity as a result of Cu doping was observed over the entire temperature sweep range, and the reproducibility of the temperature dependence was confirmed by the ramp-down and ramp-up tests. These facts demonstrate the high stability of the metal nanoparticle surface decoration doping. The low thermally excited noise level appearing at room temperature was eliminated at low temperatures in the most conductive nanosheet. Figure 3d demonstrates the change in temperature-dependent conductivity in the other nanosheet tested, with additional Ag doping. To directly observe the changes originating from the different doping processes, we calculated the normalized conductivity at room temperature, using the following equation: *σ*_normalized_ = (*σ_T_* − *σ_T_* _= 300 K_)/*σ_T_* _= 300 K_, where *T* is the temperature. The RuO_2_ nanosheets without metallic nanoparticle surface decoration exhibited a semiconducting behavior; that is, the conductivity decreased corresponding to a decrease in temperature, because of the low carrier density due to the small band overlap, despite the semi-metallic band structure of RuO_2_ [29,30]. The charge carriers transferred by the Cu nanoparticles increased the electrical conductivity of RuO_2_ nanosheets and the temperature dependence of conductivity. However, the Cu surface treatment could not overcome the temperature-dependent decrease in intrinsic carrier density. A charge exceeding the intrinsic carrier density was transferred during the Ag surface treatment at temperatures above 250 K. As a result, at room temperature, a metallic behavior with an increase in electrical conductivity with decreasing temperature was observed [32]. As the temperature decreased below 250 K, however, it changed to a semiconducting temperature behavior, due to the decrease in intrinsic carrier density. We established that the charge density transferred by the Cu- and subsequence Ag-nanoparticle decoration process was comparable to the internal carrier density. In this nanosheet, the noise level increased with decreasing temperature, indicating unstable electric contact between the nanosheet and electrodes.

### 3.2. Contact Resistance

**Figure 4:**
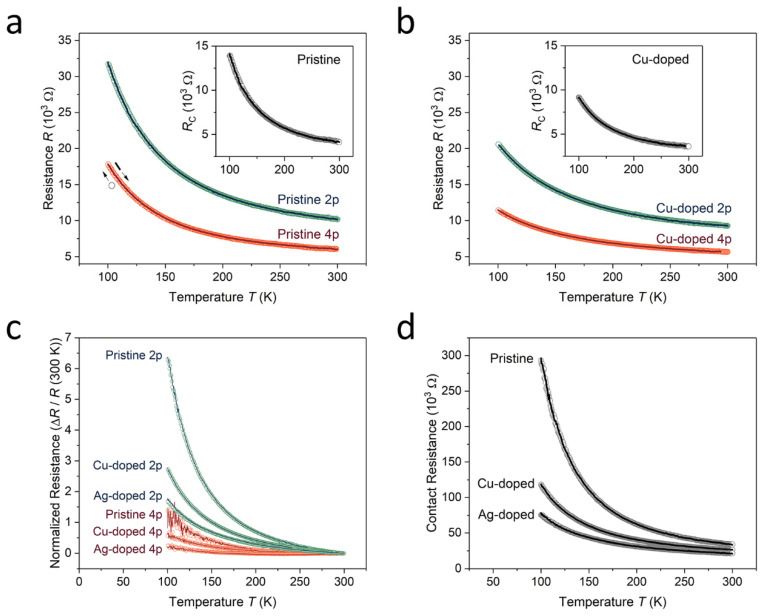
Contact resistances. (**a**) Temperature dependent resistances of a pristine monolayer RuO_2_ nanosheet measured by the four- (orange) and two-probe (green) measurement techniques. The inset image shows the contact resistances of the nanosheet as a function of the temperature obtained from the two different measurement configurations. The open circles and solid lines indicate the data measured during temperature ramp-down and ramp-up, respectively. (**b**) Temperature dependence of the four-probes (orange), two-probes (green), and contact resistances (inset) obtained from the nanosheet after the surface decoration of Cu-nanoparticles. (**c**) Direct comparison of the temperature dependencies of the four- (orange) and two-point probe (green) measurements in pristine, Cu-doped, and Ag-doped states using the normalized resistances at room temperature. (**d**) Change of temperature-dependent contact resistance during the doping processes calculated from the two measurement configurations.

Figure 4a demonstrates the raw data measured by the four- and two-point probe techniques at a 10 nA current for the pristine monolayer RuO_2_ nanosheet with the highest conductivity. The high reproducibility and low noise level, despite the infinitesimal measured, current indicated the stability of the electrical contact between the nanosheet and metal electrodes formed by the plasma etching process. We determined the contact resistance values by calculating the difference between the resistances of the two measurement configurations. The contact resistance in the pristine nanosheet increased from about 4.1 to 14.0 kΩ, corresponding to a decrease in temperature from 300 to 100 K, and accounted for approximately 40% of the total device resistance. The surface decoration with metal nanoparticles marginally reduced the semiconducting properties of the pristine RuO_2_ nanosheet. As shown in Figure 4b and inset, the Cu doping effect decreased the temperature dependence of resistance and decreased the contact resistance, to approximately 3.6 kΩ at 300 K and 9.1 kΩ at 100 K. The proportion of contact resistance in the total device resistance was approximately equal to the proportion of contact resistance before Cu doping. We observed that the proportion remained unchanged (a deviation of 0.6%) at low temperatures. This was because of the increased sensitivity of the carrier density dependence owing to the decreased mobility change at low temperature [29]. The ratios of the resistance of doped nanosheets to the resistance of pristine nanosheets remained equal in both measurement configurations at 100 K. Moreover, the ratios of the contact resistance to the total resistance remained approximately the same (deviation less than 1% at 100 K and 3% at room temperature) after the doping process. These facts suggest that the contact resistance of RuO_2_ nanosheets is determined by the carrier density over the entire temperature sweep range.

We demonstrated these phenomena of the contact resistance in the Ag treatment with a higher doping effect. As shown in Figure 4c, the semiconducting properties of the RuO_2_ nanosheets decreased during each doping process. This phenomenon was represented using the normalized resistance calculated from the following equation: *R*_normalized_ = (*R_T_* − *R_T_* _= 300 K_)/*R_T_* _= 300 K_. The contact resistances were successfully extracted from the noisy raw data of the four-probe resistances by fitting the measured data (Figure 4d). As shown in the most conductive nanosheet, the Cu nanoparticles decreased the contact resistance in this nanosheet with low conductivity, and an additional reduction was demonstrated through subsequent Ag nanoparticle decoration. In contrast, the contact resistance values of this nanosheet were found to be five-fold larger than that of the most conductive nanosheet. This is because of the incomplete surface treatment before the metallization of the electrodes, leading to an increase in the total resistance, as well as the amplified noise, by degrading the benefits of the four-probe technique.

## 4. Conclusions

We analyzed the changes in electrical conductivity and contact resistance of RuO_2_ nanosheets using charge transfer doping effects based on surface metal nanoparticle decoration. The electrical conductivity of RuO_2_ nanosheets increased gradually because of the surface decoration with Cu- and Ag nanoparticles. The additional carriers transferred from the metal nanoparticles to the RuO_2_ nanosheets decreased the semiconducting temperature behavior of the pristine RuO_2_ nanosheets, due to insufficient carrier density. We obtained the contact resistance between the nanosheet and electrodes from the resistances measured using the four- and two-point probe techniques. Moreover, the contact resistance decreased gradually during the surface decoration processes. From a quantitative analysis based on the two different measurement configurations, we observed that the resistance and carrier density of the nanosheets had a significant impact on the contact resistance. The surface decoration of metal nanoparticles for external contact engineering, as well as the modulation of the internal properties of nanomaterials, can be successfully used for future research in the field of 2D materials.

## Data Availability

Data are contained within the article.
